# List No. 5 of Training-Schools of Registered Nurses

**Published:** 1918-03-16

**Authors:** 


### THE COLLEGE OF NURSING

(Limited by Guarantee).

### List No. 5 of Training-Schools of Registered Nurses.

At the last Council meeting of the College of Nursing applicants for membership were passed holding certificates of the following training-schools :

Charing Cross Hospital, W.C.; Guy's Hospital, S.E.; King's College Hospital, S.E.; London Hospital, E.; The London Temperance Hospital, N.W.; Middlesex Hospital, W.; Poplar Hospital, E.; Royal Free Hospital, W.C.; St. George's Hospital, S.W.; St. John's Hospital, Lewisham; St. Mary's Hospital, Paddington; St. Thomas's Hospital, S.E.; West London Hospital, Hammersmith; Bermondsey Poor-Law Infirmary; Bethnal Green Union Infirmary; Central London Sick Asylum, Hendon; Edmonton Poor-Law Infirmary, N.; Hackney Union Infirmary, Homerton; Kingston Poor-Law Infirmary, Kingston-on-Thames; Lewisham Poor-Law Infirmary; St. Marylebone Infirmary, London; Mile End Poor-Law Infirmary, E.; St. Giles' Poor-Law Infirmary, Camberwell; St. John's Hill Infirmary, Wandsworth; St. Pancras North Union Infirmary, N.; Shoreditch Union Infirmary, London; Woolwich Union Infirmary, S.E.; General Hospital, Tunbridge Wells, Kent; Addenbrooke's Hospital, Cambridge; District Infirmary, Ashton-under-Lyne; North Devon Infirmary, Barnstaple; Queen's Hospital, Birmingham; Blackburn and East Lancs Royal Infirmary; Infirmary and Dispensary, Bolton; Bootle Borough Hospital, Liverpool; Royal Sussex County Hospital, Brighton; Royal Infirmary, Bristol; Royal Infirmary, Chester; Guest Hospital, Dudley; Royal Infirmary, Halifax; Hartlepools Hospital, Hartlepool; St. Helen's Hospital, Peaseley Cross, Lancs; Royal Infirmary, Huddersfield; Kettering and District General Hospital; Warneford Hospital, Leamington; General Infirmary, Leeds; Royal Infirmary, Leicester; Royal Infirmary, Liverpool; Royal Southern Hospital, Liverpool; Stanley Hospital, Liverpool; Royal Infirmary, Manchester; Salford Royal Hospital; Royal Victoria Infirmary, Newcastle-on-Tyne; Norfolk and Norwich Hospital, Norwich; General Hospital, Nottingham; Royal Hospital, Portsmouth; South Devon and East Cornwall Hospital, Plymouth; Royal Berkshire Hospital, Reading; Royal Isle of Wight County Hospital, Ryde; Royal Infirmary, Sheffield; Stockton and Thornaby Hospital, Stockton-on-Tees; Wolverhampton and Staffordshire General Hospital; General Infirmary, Worcester; Wrexham Infirmary, North Wales; Birmingham Poor-Law Infirmary; Selly Oak Poor-Law Infirmary; Union Infirmary, Blackburn; Townleys Poor-Law Hospitals, Bolton; St. Luke's Poor-Law Hospital, Halifax; Anlaby Road Poor-Law Infirmary, Hull; Keighley Union Infirmary; Leeds Poor-Law Infirmary, Beckett Street; Brownlow Hill Poor-Law Infirmary, Liverpool; Liverpool Union Infirmary; Withington Poor-Law Hospitals, West Didsbury; Crumpsall Poor-Law Infirmary, Manchester; Middlesbrough Union Infirmary; Newcastle-on-Tyne Union Hospital; Sheffield Union Hospital, Fir Vale; Stoke-on-Trent Union Infirmary; Queen Mary's Hospital, Stratford, E.; St. Bartholomew's Hospital, E.C.; University College Hospital, W.C.; Westminster Hospital, S.W.; Fulham Poor-Law Infirmary; Holborn Union Infirmary, Highgate; Lambeth Poor-Law Infirmary; Paddington Poor-Law Infirmary; Poplar and Stepney Sick Asylum, E.; St. James' Poor-Law Infirmary, Wandsworth; St. George-in-the-East Poor-Law Infirmary, E.; West Ham Union Infirmary, Leytonstone; General Hospital, Birmingham; Kent and Canterbury Hospital, Canterbury; King Edward VII.'s Hospital, Cardiff; Royal West Sussex Hospital, Chichester; Royal Infirmary, Derby; Royal Albert Hospital, Devonport; West Herts Hospital, Hemel Hempstead; Royal Infirmary, Hull; Monkwearmouth and Southwick Hospital, Sunderland; Royal Infirmary, Oldham; General Infirmary, Salisbury; Royal Hospital, Sheffield; North Staffs Infirmary, Stoke-on-Trent; Royal Hants County Hospital, Winchester; West Bromwich District Hospital, Staffs; County Hospital, York; Ashton-under-Lyne Union Hospitals; Erdington Poor-Law Infirmary, Birmingham; Poor-Law Infirmary, Coventry; Union Infirmary, Kingston-on-Thames; Mill Road Poor-Law Infirmary, Liverpool; Union Infirmary, Merthyr Tydvil; West Derby Union Infirmaries, Walton; Bagthorpe Poor-Law Infirmary, Nottingham; Stepping Hill Poor-Law Hospital, Stockport; Shirley Warren Poor-Law Infirmary, Southampton; Seamen's Hospital, Greenwich and Royal Waterloo Hospital, S.E. 1; Brompton Hospital, S.W., and St. Thomas's Hospital, S.E. 1.

